# Altered Lipid Profiles and Vaccine Induced-Humoral Responses in Children Living With HIV on Antiretroviral Therapy in Tanzania

**DOI:** 10.3389/fcimb.2021.721747

**Published:** 2021-11-09

**Authors:** Wilbert Mbuya, Issakwisa Mwakyula, Willyelimina Olomi, Peter Agrea, Francesco Nicoli, Cecilia Ngatunga, Leodegard Mujwahuzi, Paul Mwanyika, Mkunde Chachage

**Affiliations:** ^1^ National Institute for Medical Research (NIMR), Mbeya Medical Research Centre (MMRC), Mbeya, Tanzania; ^2^ Department of Internal Medicine, Mbeya Zonal Referral Hospital and University of Dar es Salaam Mbeya College of Health and Allied Sciences, Mbeya, Tanzania; ^3^ Department of Chemical, Pharmaceutical and Agricultural Sciences, University of Ferrara, Ferrara, Italy; ^4^ Department of Radiology, Mbeya Zonal Referral Hospital and University of Dar es Salaam Mbeya College of Health and Allied Sciences, Mbeya, Tanzania; ^5^ Department of Paediatric, Mbeya Zonal Referral Hospital and University of Dar es Salaam Mbeya College of Health and Allied Sciences, Mbeya, Tanzania; ^6^ Department of Microbiology and Immunology, University of Dar es Salaam - Mbeya College of Health and Allied Sciences (UDSM-MCHAS), Mbeya, Tanzania

**Keywords:** HIV, children, dyslipidemia, cardiovascular disease, immune senescence, childhood vaccines, ART

## Abstract

People living with HIV, even under therapy, have a high burden of age-related co-morbidities including an increased risk of dyslipidemia (which often predisposes to cardiovascular diseases) and immune-aging. In this study, lipid profiles and antibody responses to measles and pertussis toxin vaccines were compared between ART experienced HIV+ children (n=64) aged 5-10 years, and their age- and sex-matched HIV- controls (n=47). Prevalence of high-density lipoprotein cholesterol (HDL-c) and triglyceride-driven dyslipidemia was higher among treated HIV+ children than in controls (51.6% vs 27.7% respectively, p < 0.019). In a multivariate Poisson regression model adjusted for age, sex and BMI, the association between low HDL-c, hypertriglyceridemia and HIV remained significantly high (for HDL-c: ARR: 0.89, 95% CI: 0.82 – 0.96, p = 0.003; for triglycerides: ARR: 1.54, 95% CI: 1.31 – 1.81, p < 0.001). Among HIV+ children, the use of lopinavir/ritonavir, a protease-based antiretroviral therapy was also associated elevation of triglyceride levels (p = 0.032). Also, HIV+ children had a 2.8-fold reduction of anti-measles IgG titers and 17.1-fold reduction of anti-pertussis toxin IgG levels when compared to HIV- children. Our findings suggest that dyslipidemia and inadequate vaccine-induced antibody responses observed in this population of young African HIV+ children might increase their risk for premature onset of cardiovascular illnesses and acquisition of preventable diseases.

## Introduction

Despite the importance of ART in reducing HIV morbidity and mortality, people living with HIV (PLWH) have, even under therapy, increased morbidity and decreased life expectancy (Antiretroviral Therapy Cohort Collaboration et al., 2009; Modrich et al., 2010; IeDEA Pediatric Working Group, 2017). Furthermore, compared to the general population, PLWH have an accelerated aging process, which is often associated with a high burden of age-related co-morbidities (Deeks and Phillips, 2009; Guaraldi et al., 2011).

There is a growing body of literature that recognizes an increased risk of age-related conditions such as cardiovascular diseases (CVD) for HIV positive persons at a younger age compared to that of their age-matched uninfected counterparts (Maggi et al., 2017). Several studies have documented a higher prevalence of traditional CVD risk factors such as obesity, diabetes, smoking and dyslipidemia in PLWH (Rahmanian et al., 2011; Tien et al., 2012; Paisible et al., 2015). HIV-associated dyslipidemia is often characterized by elevated levels of triglycerides and/or heightened low-density lipoprotein cholesterol (LDL-c) levels and reduced high-density lipoprotein cholesterol (HDL-c). Certain types of ART also contribute to a high prevalence of dyslipidemia in PLWH (Nduka et al., 2015), further contributing to increased risk of CVD. Apart from dyslipidemia and other traditional CVD risk factors, long-term ART exposure and HIV-induced chronic inflammation and immune activation that persists despite the use of ART may also drive cellular aging and thus contribute to increasing the risk for CVD in HIV+ people (McDonald and Kaltman, 2009; Syed et al., 2013; Maggi et al., 2017).

Atherosclerosis begins in childhood, prospectively increasing the risk of developing CVD (Daniels et al., 2011; Jaquith et al., 2013), indicating the importance of characterizing the impact of HIV and ART on CVD already at an early age. Indeed, HIV+ children are projected to display mortality rates 30 times higher than uninfected children due to age-related disorders (Brady et al., 2010; Guaraldi et al., 2011; Jaquith et al., 2013; Chiappini et al., 2014; Guaraldi and Palella, 2017). Therefore, it is plausible to think that the accelerated aging process described in adults affects children living with HIV as well. Currently, 1.8 million children live with HIV infection worldwide, with the vast majority of them residing in Africa (Global Statistics | HIV.Gov n.d.). In Tanzania, an estimated 93,000 children are living with HIV and 66% of them receiving ART (Global Statistics | HIV.Gov n.d.). In the absence of a cure, these children are set to receive ART throughout their lives and are thus vulnerable to HIV-mediated age-related illnesses which will not only impact their quality and length of life but will also overwhelm the already burdened healthcare systems of most resource-limited countries like Tanzania.

The accelerated aging experienced by HIV+ individuals also affects the immune system (Appay et al., 2007; Pathai et al., 2013), causing exhaustion of B and T immune cells, and inadequate response to vaccines (Pinti et al., 2016). Conditions associated with premature aging, including CVD risk, dyslipidemia and immune-aging have been poorly characterized in HIV+ African children (Gianesin et al., 2016), even though these illnesses may seriously threaten the future of these children. Hence, a need to identify and monitor for risk factors associated with CVD and immunoscenescence early on HIV+ children, even when they have suppression of viral replication and normal CD4 T cell counts. Therefore, this study sought to characterize premature aging in ART-treated HIV positive children focusing on lipid metabolic abnormalities as a risk factor for CVD, immune aging, and to understand their mutual relationship.

## Materials and Methods

### Study Population and Ethical Consideration

One hundred and eleven age- and sex- matched HIV+ and HIV- female and male children aged between 5 and 10 years were recruited into the study from June to December 2020 in Mbeya, Tanzania. HIV+ children (n=64) were recruited from routine care and treatment pediatrics clinic at the Mbeya Referral Zonal Hospital (MZRH) in Mbeya, Tanzania. Healthy controls (n=47) included children who had previously been treated for acute conditions at MZRH, children known by hospital staff and siblings/relatives (n=2) of enrolled HIV+ children. This study was reviewed and approved by the Mbeya Medical Research and Ethics review Committee (SZEC-2439/R.A/V.1/61). Written Informed consent or assent was obtained from all parents/legal guardians and study volunteers and (when necessary) prior to any study related interview, clinical evaluation or sample acquisition.

### Clinical Assessments and Specimen Collection

Interviews, clinical examination of the children and sample collection for laboratory assays were conducted by certified nurse and clinicians, and in accordance with the study approved protocol and national medical guidelines. A review of ART history, past and current medical diagnoses as well as demographics was carried out on HIV+ children using a questionnaire. Information on demographics and medical history was also collected from controls. Clinical examination included blood pressure and pulse rate measurements, height, weight and evaluation of participants’ general health. For laboratory assays, peripheral blood was collected by venipuncture into SST tube (BD) for HIV rapid testing and clinical chemistry measurements, and into EDTA tubes (BD) for plasma HIV viral load and absolute CD4 T-cell quantification. Plasma obtained from the whole blood was used to quantify IgG antibody titers against measles and pertussis toxin and systemic IL-6 levels by Enzyme linked Immunosorbent Assay (ELISA). Immediately upon collection, the samples were transported to the National Institute for Medical Research – Mbeya centre main laboratory.

### Clinical Chemistry (Triglycerides, HDL-c, LDL-c and Total Cholesterol)

Clinical chemistry parameters in this study were measured by Cobas integra 400 (Roche). Normal reference ranges used were established by the National Institute for Medical Research – Mbeya Medical Research Centre (NIMR-MMRC) for children living in Mbeya region, Tanzania (**Supplementary Table 1**). Lipid abnormalities/dyslipidemia were defined as having either or a combination of: Triglycerides > 2.18 mm/L in males and >2.88 mm/L in females; HDL-c < 1.1 mm/L in both males and females; LDL-c > 4.3 mm/L in both males and females; total cholesterol > 5.5 mm/L in both males and females.

### HIV Testing, ART Usage Information, CD4 Cell Counts and HIV Viral Load Quantification

HIV status was determined by SD Bioline (Standard Diagnostics) and if positive confirmed by Unigold (Trinity Biotech). For HIV+ children, CD4 counts were quantified by flow cytometry using either BD Facs Canto™ II (BD) or Facs Calibur™ (BD). HIV plasma viral loads were quantified by Xpert HIV-1 Viral Load kit (Cepheid). Information on ART was obtained by interviewing guardians of HIV+ volunteers and from the hospital database. As per the current Tanzanian national guidelines (The United Republic of Tanzania, 2019), preferred first line ART regimen for children’s with <20kg is a protease inhibitor (PI)-based regimen containing lopinavir and ritonavir (LPV/r) with nucleoside reverse transcriptase inhibitors (NRTI) combinations: abacavir and lamivudine (ABC/3TC). Integrase inhibitor, dolutegravir (DTG)-based regimen in combination with ABC and 3TC is recommended as the preferred 1^st^ line for children living with HIV weighing ≥20kg. Previously (The United Republic of Tanzania, 2017), the following drug combinations were recommended as 1^st^ line treatment for children:

- ABC/3TC+LPV/r or zidovudine (AZT)/3TC+LPV/r
- Non-nucleoside reverse transcriptase inhibitor (NNRTI) regimen containing nevirapine (NVP) in combination with NRTIs, AZT/3TC
- NNRTI regimen containing efavirenz (EFV) with one of the following NRTIs: AZT/3TC or ABC/3TC or tenofovir (TDF)/3TC

### ELISA for Human IL-6, IgG for Measles and Pertussis

From sera samples, systemic human IL-6 levels were quantified by Human IL-6 ELISA kit (Invitrogen), anti-measles IgG titers were quantified by Measles IgG ELISA kit (Alpha Diagnostics) while anti-B. pertussis toxin IgG titers were quantified by Human anti-B. Pertussis Toxin/Toxoid (PTX) IgG ELISA kit (Alpha Diagnostics). For all three kits, manufacturers instruction were followed. Sunrise ELISA reader (Tecan) was used to measure the absorbance of the samples after the assays. Standards were used to determine the concentration of the respective analyte in relation to optical density determined by a plate reader. As per kit instructions, IgG titers were grouped as follows: For measles, below 8 U/ml was termed as negative, 8 U/ml to 12 U/ml was termed as equivocal and above 12 U/ml was regarded as a positive response. For pertussis, below 18 U/ml was termed as negative, 18 U/ml to 22 U/ml was termed as equivocal and 22 U/ml and above was regarded as a positive response. Cytokine and antibody concentrations below or above the measurable range were reported as the minimum or maximum measurable values respectively.

### Statistical Analysis

Stata version 16 (StataCorp, USA) and GraphPad Prism software version 9 (GraphPad Software Inc, USA) were used for statistical analysis. Two tailed Mann-Whitney U test was done to compare the quantities of lipids and plasma levels of human IL-6, Measles and Pertussis IgG titers when stratified by HIV status or by ART-based regimens. Fisher’s exact test was used to compare the proportion of different lipid clinical ranges between HIV+ and HIV- children. Spearman’s rank correlation test was used to assess correlation between different variables. Multivariable poisson regression models were used to adjust for confounders, 95% confidence interval and p-value was reported. Statistical significance was defined as p < 0.05.

## Results

### Description of the Study Participants

A total of 111 children aged between 5 and 10 years were recruited into the study. Of these, 64 were HIV+ while 47 were HIV-. Groups were age- and sex-matched and had similar BMI and blood pressure measurements (**Table 1**). Within the HIV+ group, the median age at HIV diagnosis was 2.5 years. All of the HIV+ children were on ART with a median CD4 count of 887.5 cells/mm^3^ and median plasma viral load copies of 39 copies/ml at the time of enrollment. At enrollment, 42% of children living with HIV (CLWH) were on protease inhibitor (PI) based regimen while 50% were on Integrase inhibitor, dolutegravir (DTG)-based regimen and 8% were on NNRTI based regimen. Of note, 22% (7/32) and 53% (17/32) of HIV+ children who are currently taking DTG-based regimen had previously been on PI- and NNRTI based ART regimens respectively. These were shifted to DTG therapy a median of 5.5 months ago. **Table 1** details the demographic and clinical characteristics of study participants.

**Table 1:** Clinical parameters of the study participants.

	HIV Negative (n = 47)	HIV Positive (n = 64)	p-value^a^
Age (years)	7 (6 -9)	7.5 (6 -8.5)	0.576
Gender (% male)	57.5	50	0.449*
Median age at HIV Diagnosis (years)		2.5 (1.2-5.8)	
CD4 counts (cells/ul)		887.5 (620 - 1426)	
HIV viremia (copies/mL)		39 (0 – 39)	
Duration of ART Usage (months)		57.5 (13.6 – 76.6)	
%PI - Lopinavir/Ritonavir		42.2	
BMI (kg/m^2^)	15.5 (14.8 – 16.1)	15.6 (15.1 – 16.6)	0.134
Weight (kg)	21.2 (19 – 24.8)	21.2 (19 – 24.3)	0.745
Height (cm)	118 (112 - 124)	116.7 (110.1 – 122.2)	0.137
Respiratory rate (beats/minute)	22 (22 - 24)	22 (21 - 24)	0.835
Systolic Blood Pressure (mmHg)	100 (100 - 102)	102 (100 - 111)	0.184
Diastolic Blood Pressure (mmHg)	68 (62 - 72)	69 (62 - 72)	0.812

All data are median (IQR) unless otherwise specified.

a Mann-Whitney U-test unless otherwise specified.

*Fishers exact test.

### Reduced HDL-c and Elevated Triglyceride Levels in HIV+ Children

**Figure 1:**
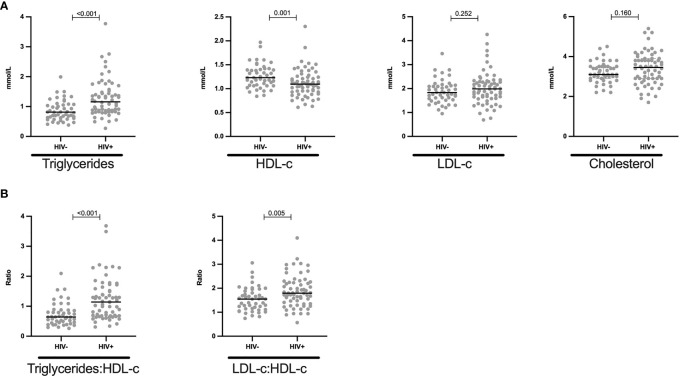
Reduced HDL-c and increased triglycerides levels in HIV+ children: **(A)** Plasma quantity for triglycerides, HDL-c, LDL-c and cholesterol is shown in mmol/L and stratified by HIV status. **(B)** Triglycerides-HDL-c ratio and LDL-c-HDL-c ratio is shown in HIV negative and HIV positive children. Each dot represents an individual volunteer, the median is indicated. Statistical analysis was performed using the Mann-Whitney U-test.

To determine the effect of HIV on lipid plasma levels, we stratified levels of cholesterol, triglycerides, HDL-c and LDL-c by HIV status. The prevalence of dyslipidaemia (as defined in the methods and **Supplementary Table 1**) was higher in HIV+ than of HIV- children [51.6% (33/64) vs 27.7% (13/47) respectively, p = 0.019, **Table 2**]. Specifically, 50% of CLWH had abnormally low concentrations of HDL-c while 27.7% (13/47) of controls displayed such HDL-c abnormalities (p = 0.02, **Table 2**). The prevalence of hypertriglyceridemia was 3.1% (2/64) vs 0% (0/47) for HIV+ and HIV- children respectively (p = 0.507, **Table 2**). There were no individuals with abnormal levels of cholesterol and LDL-c in this study population (**Table 2**). We observed elevated triglycerides levels (median mmol/L: 1.16 vs 0.81, p < 0.001) but reduced HDL-c levels (median mmol/L: 1.1 vs 1.23, p < 0.001) in HIV+ when compared to HIV- children respectively (**Figure 1A**). Total cholesterol (median mmol/L: 3.4 vs 3.1, p =0.197) and LDL-c (median mmol/L: 2.0 vs 1.8, p = 0.264) plasma levels were not significantly higher in this study population of treated CLWH than in the controls (**Figure 1A**). Median triglycerides-HDL-c and LDL-c-HDL-c ratios were higher in HIV+ than HIV- children, 1.14 vs 0.64, p < 0.001 and 1.5 vs 1.8, p = 0.005, respectively (**Figure 1B**).

**Table 2:** Prevalence of dyslipidaemia in HIV- and HIV+ children.

Lipid Profile, n (%)	HIV- (n = 47)	HIV+ (n = 64)	p value (Fishers exact test)
Overall dyslipidemia	13 (27.7%)	33 (51.6%)	0.019
Low HDL-c	13 (27.7%)	32 (50.0%)	0.02
Hypertriglyceridemia	0 (0%)	2 (3.1%)	0.507

Similarly, Poisson regression model showed that HIV+ children had 48% relative risk (RR) increase in triglycerides when compared to HIV- children (CRR: 1.48, 95% CI: 1.01 – 2.15, p = 0.043) and when adjusted for age, sex and BMI, we observed a 54% RR increase in triglycerides among the HIV+ children (ARR: 1.54, 95% CI: 1.31 – 1.82, p < 0.001, **Table 3**). On the other hand, a 12% RR in HDL-c reduction was found among the HIV+ when compared to HIV- children (CRR: 0.88, 95% CI: 0.63 – 1.24, p = 0.476). After adjusting for confounders, there was an 11% RR for decreased HDL-c levels among the HIV+ (ARR: 0.89, 95% CI: 0.82 – 0.96, p = 0.003, **Table 3**).

**Table 3:** Multivariate Poisson regression modelling of association of dyslipidemia and HIV infection.

Parameter	CRR	95% CI	p-value	ARR	95% CI	p-value
**Triglycerides**						
HIV-						
HIV+	1.48	1.01 - 2.15	0.043	1.54	1.31 - 1.82	< 0.001
**HDL-c**						
HIV-						
HIV+	0.88	0.63 - 1.24	0.476	0.89	0.82 - 0.96	0.003
**LDL-c**						
HIV-						
HIV+	1.07	0.82 - 1.40	0.619	1.08	0.96 - 1.20	0.205
**Cholesterol**						
HIV-						
HIV+	1.06	0.86 - 1.31	0.564	1.08	1.00 - 1.16	0.054

CRR, Crude risk ratio; ARR, Adjusted risk ratio; CI, Confidence Interval; HDL-c, High density lipoprotein – cholesterol; LDL-c, Low density lipoprotein – cholesterol

The model was adjusted for age, sex and BMI.

Together, these data indicate that HIV+ children have increased triglyceride and decreased HDL-c levels, and thus present with dyslipidaemia.

### Comparable IL-6 Plasma Levels in HIV+ and HIV- Children

**Figure 2:**
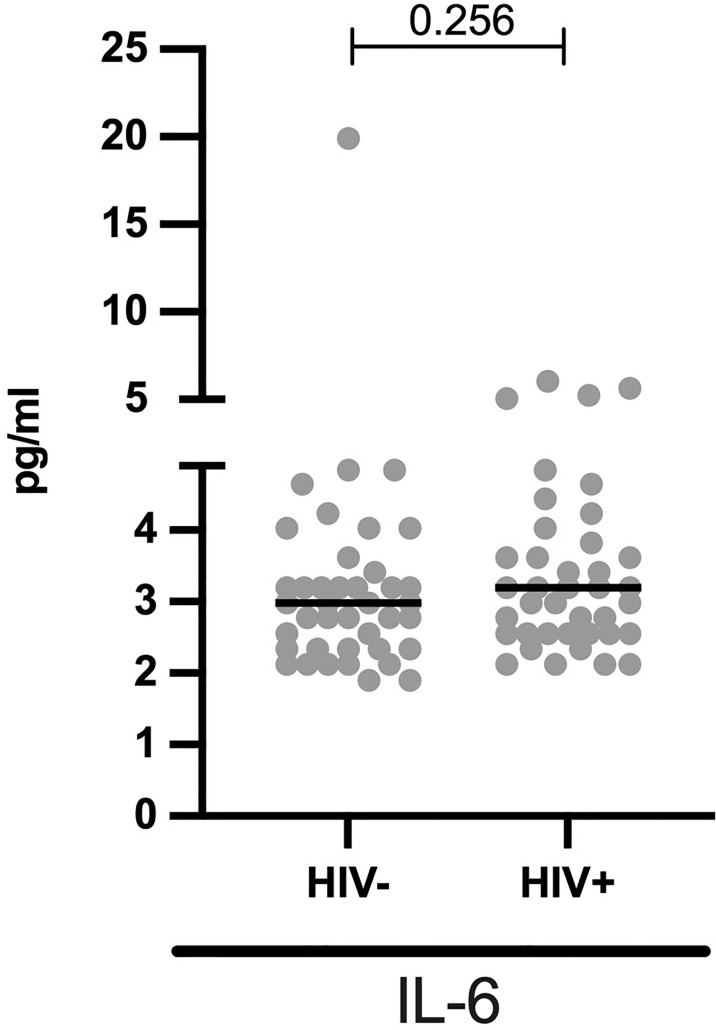
Comparable systemic IL-6 levels in HIV- and HIV+ children: Concentrations of IL-6 (pg/ml) are stratified by HIV status. Each dot represents an individual volunteer, the median is indicated. Statistical analysis was performed using the Mann-Whitney U-test.

We next measured IL-6 plasma levels (n = 79) to determine the effect of HIV on systemic inflammation in ART experienced children (57 months median ART usage). We observed that the median IL-6 levels are comparable (median pg/ml: 3.2 vs 3.0, p = 0.256) between HIV+ and HIV- children (**Figure 2**). Among HIV+ children, there was no correlation between ART duration and IL-6 plasma levels (Spearman r =0.006, p = 0.948, **Supplementary Figure 1**).

### Reduced Measles and Pertussis IgG Plasma Levels in HIV+ Children

**Figure 3:**
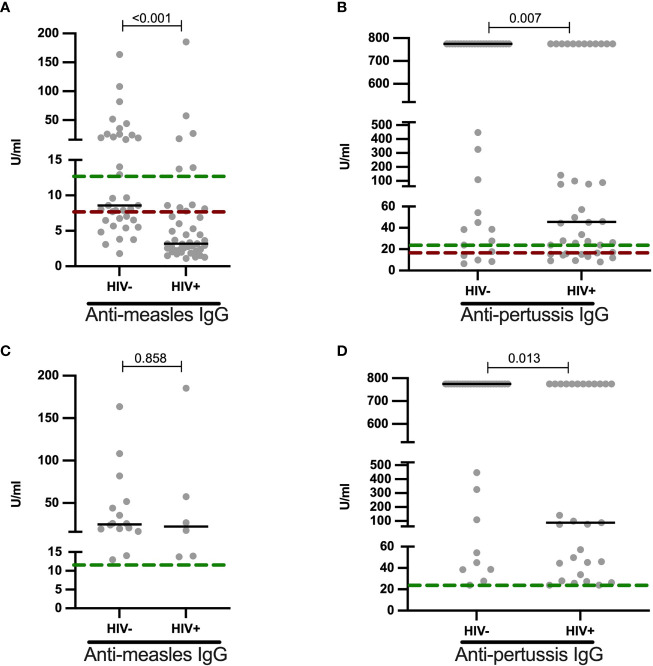
Reduced anti-measles and anti-pertussis plasma IgG titers in HIV+ children: **(A)** Anti-measles (U/ml) and **(B)** anti-pertussis (U/ml) IgG titers are shown for HIV negative and HIV positive children. Individuals with positive **(C)** anti-measles (U/ml) and **(D)** anti-pertussis (U/ml) IgG titers are shown and stratified by HIV status. Titers above green dotted line are termed as positive, those between green and red dotted lines as equivocal and those below the red line as non-responses. Each dot represents an individual volunteer, the median is indicated. Statistical analysis was performed using the Mann-Whitney U-test.

To determine the effect HIV on childhood immunizations, we measured anti-measles (n = 80) and anti-pertussis (n = 77) IgG titers in vaccinated children stratified by HIV status (**Figure 3**). According to the kits used, the results were grouped into either negative, equivocal or positive titer levels. The prevalence of positive measles vaccine-induced IgG titers was lower in HIV+ than HIV- children [(14% (6/43) vs 43% (16/37) p = 0.005, **Table 4**]. The proportion of positive IgG responses against pertussis toxin was 72.5% (29/40) in HIV+ and 86.5% (32/37) HIV- children (p = 0.165, **Table 4**). In terms magnitude, HIV+ children, though on ART, had 2.8-fold reduced anti-measles IgG levels (**Figure 3A**) and 17.1-fold reduced anti-pertussis IgG levels (**Figure 3B**) when compared to HIV- children. Additionally, we observed an inverse correlation between age and anti-measles IgG titers in CLWH but not in HIV- children (Spearman r = -0.416, p = 0.006 for HIV+ and Spearman r = -0.241, p = 0.150 for HIV-, **Supplementary Figure 2**). In samples with positive anti-measles and anti-pertussis IgG titers, median anti-measles IgG titers were comparable between HIV+ and HIV- volunteers (median: 22.32 U/ml vs 24.86 U/ml, p value=0.858, **Figure 3C**) but an 8.6-fold reduction in median anti-pertussis IgG titers was observed in HIV+ children (median: 88.59 U/ml for HIV+ vs 775.2 U/ml for HIV-, p value=0.013, **Figure 3D**). This indicates a reduced humoral response to particularly anti-pertussis vaccine in these CLWH despite being on ART.

**Table 4:** Prevalence of anti-measles and anti-pertussis IgG responders in HIV- and HIV+ children.

Antibody, n (%)	HIV-	HIV+	p value (Fishers exact test)
Anti-Measles	16 of 37 (43.2%)	6 of 43 (14.0%)	0.005
Anti-Pertussis	32 of 37 (86.5%)	29 of 40 (72.5%)	0.165

### Elevated Triglycerides and Cholesterol Levels in HIV+ on Lopinavir/Ritonavir Associated Inhibitors ART Regimen

**Figure 4:**
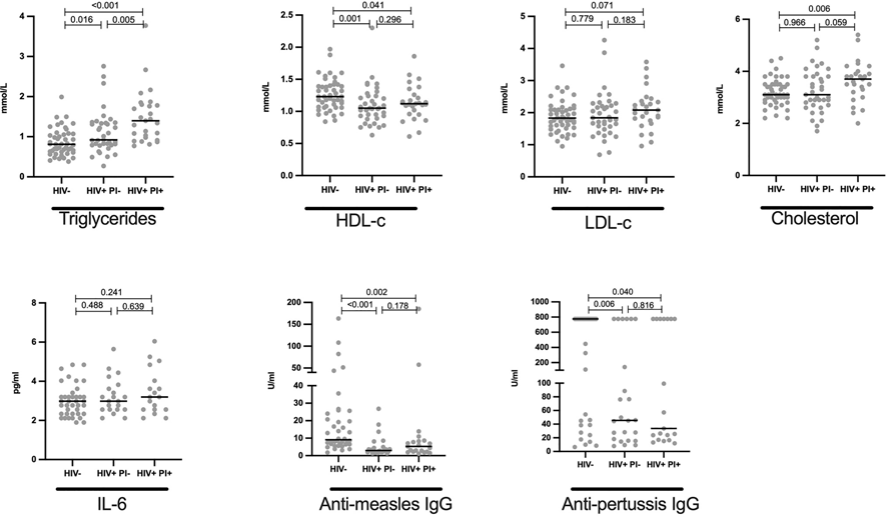
Elevated levels of cholesterol and triglycerides in HIV+ children on protease inhibitor ART: Plasma quantity for triglycerides, HDL, LDL, cholesterol in mmol/L; IL-6 in pg/ml, anti-measles IgG and anti-pertussis in U/ml are shown and stratified by HIV status. Each dot represents an individual volunteer, the median is indicated Statistical analysis was performed using the Mann-Whitney U-test.

To determine the effect of PI, lopinavir/ritonavir on lipid profile of HIV+ children, we stratified data by HIV status and subsequently by lopinavir/ritonavir usage. We herein report that, among the HIV+, triglyceride levels were slightly elevated in children on lopinavir/ritonavir (median mmol/L: 1.37) compared to those off lopinavir/ritonavir (median mmol/L:1.03, p = 0.032, **Figure 4**). Similarly, cholesterol was increased in children on PI-based regimen when compared to those on DTG based treatment (median mmol/L: 3.6 vs 3.2, p = 0.125, **Figure 4**). Lopinavir/ritonavir did not affect plasma HDL-c and LDL-c levels in the studied population (**Figure 4**). Similarly, PI therapy was not associated with changes in IL-6, anti-measles and anti-pertussis IgG levels (**Figure 4**).

### Association of Lipid Profiles With Immunity to Childhood Vaccines and Inflammation

**Figure 5:**
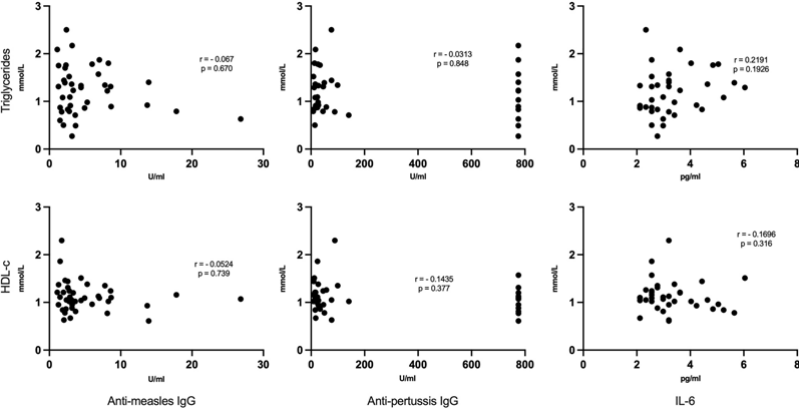
No correlation between lipid levels and anti-measles, anti-pertussis plasma IgG titers or IL-6: Each dot represents respective lipid [triglycerides (upper panels) and HDL-c (lower panels)] and vaccine titers/IL-6 serum levels. Statistical analysis was performed using the Spearman’s rank correlation test.

We found no significant correlation between lipid levels (HDL, triglycerides, LDL and cholesterol) and anti-measles and anti-pertussis titers (**Figure 5**). Similarly, no notable association was observed between each lipid and IL-6 (**Figure 5**).

## Discussion

People living with HIV with access to antiretroviral therapy (ART) are living longer, yet have significantly increased risk for age-related illnesses including cardiovascular disorders. Premature aging and risk for cardiovascular disease (CVD) is understudied in children living with HIV, even though it is well known that subclinical CVD can begin in early childhood. Indeed, we observed that, 51.6% of CLWH had dyslipidemia compared to 27.7% of HIV- children. The prevalence of dyslipidemia was largely attributed to a higher proportion of abnormally low HDL-c and increased levels of triglycerides in HIV+ children. These observations were further confirmed in a multivariate regression analysis, after adjusting for confounders. Our findings are comparable to prior studies that have analysed blood lipid disorders in adolescents and adults living with HIV living in similar settings (Ombeni and Kamuhabwa, 2016; Fiseha et al., 2021; Hamooya et al., 2021; Tilahun et al., 2021). A recent Zambian cross-sectional study found a high prevalence (63%) of low HDL-c in ART experienced young adults living with HIV aged 18-24 years and that these young individuals were about three times more likely to have low HDL-c than older individuals (Hamooya et al., 2021). Also similar to our findings, the prevalence of low HDL-c and hypertriglyceridemia has also been observed in other studies with similar settings to be higher on ART experienced HIV+ than HIV- children (Mandal et al., 2016; Dirajlal-Fargo et al., 2021). Dyslipidemia, particularly low HDL levels in treated HIV+ pediatrics has also been reported in Tanzanian and Ethiopian populations (Tadesse et al., 2019; Irira et al., 2020). However, to our knowledge, none of the earlier studies conducted in Africa have compared the level of dyslipidemia between age-matched HIV- and ART experienced HIV+ young children of less than 10 years.

While several studies have reported elevated systemic IL-6 levels in HIV+ individuals (Breen et al., 1990; Ullum et al., 1996; Shah et al., 2011; Gianesin et al., 2016), we here report similar IL-6 levels in HIV+ vs HIV- children. HIV associated factors such as duration of HIV infection, CD4 counts and HIV RNA plasma levels can affect IL-6 levels (Borges et al., 2015). We postulate that, there is no difference in IL-6 levels between the HIV+ vs HIV- children in our study because these children have been on ART for a median of 4.5 years, thus, have efficiently suppressed HIV viral replication and sufficiently restored their CD4 T -cell counts. Moreover, the children were HIV diagnosed in this *test and treat* era and so are likely to have had high nadir CD4 counts. In line with this, Borges et al. showed that IL-6 levels were increased in individuals with high HIV RNA levels with low nadir CD4 counts (Borges et al., 2015). Furthermore, Osuji and colleagues showed a 2.5 reduction in IL-6 levels just one year after ART initiation (Osuji et al., 2018). In similar recent studies, comparable levels of IL6 were observed between viral suppressing HIV+ children (from Africa and US) and their uninfected counterparts, further confirming our hypothesis (Wilkinson et al., 2018; Dirajlal-Fargo et al., 2020).

Ageing is characterized by increased dyslipidemia and blunted vaccine responses. To evaluate if such features also characterize HIV+ children, as a potential result of HIV-induced premature senescence, we measured antibody responses to childhood vaccination. In line with previous studies (Melvin and Mohan, 2003; Mutsaerts et al., 2018; Simani et al., 2019), we observed lower magnitude of antibody titers to measles and pertussis toxin in ART experienced CLWH. We also found a lower seropositivity prevalence to especially measles in CLWH on ART compared to HIV- children, suggesting inadequate measles vaccine-induced humoral response even in treated CLWH. Comparably, Pensieroso et al. observed the proportion of children from Italy with anti-measles seropositivity about 4 years post-vaccination to be 82% for HIV+ART+ children and 100% for healthy controls (Pensieroso et al., 2009). Similarly, the prevalence of measles sero-protection (measured by neutralization assay) in CLWH on ART from the US was also found to be substantially lower than that of HIV exposed uninfected children (57% vs 99%) 9 years post-vaccination (Siberry et al., 2015). Interestingly, within our cohort of HIV+ children, age was strongly associated with inadequate antibody responses to measles vaccine, further suggesting the accelerated aging processes even in ART experienced children.

Surprisingly, under half of healthy controls who reported to having received vaccination had protective IgG antibodies against measles. This is unlikely to be due to waning of protection with time, since anti-measles IgG antibodies are known to persist for decades in healthy individuals (WHO n.d.). Since Measle-Rubella (MR) vaccine is usually given at a later stage in this region (9 and 18 months of age) compared to Diptheria-Tetanus and Pertussis (DTP) containing vaccine which is given 3 times before a child turns four months, it is plausible to think that some of the children reported to having received childhood immunization may have missed some vaccines including measles vaccine. Indeed, despite having one of the highest coverage rates of routine childhood immunization programme, over a quarter of children are not fully vaccinated in Tanzania (Tanzania, 2015; Vasudevan et al., 2020).

Previous studies (mostly done in HIV+ adults) have linked the use of protease (PI)-based ART with increased dyslipidaemia (Cunha, 2015; De Lima et al., 2018; Irira et al., 2020; Belete et al., 2021; Hamooya et al., 2021; Tilahun et al., 2021). HIV+ individuals on PI-based regimen are more likely to have higher concentrations of triglycerides than those who were on non-PI therapies (Kazooba et al., 2017; Belete et al., 2021). In this study, we similarly observed that CLWH receiving protease-based therapy (lopinavir boosted with ritonavir) had moderately higher triglyceride levels than CLWH who were on dolutegravir, an integrase-based regimen. These findings suggest that dyslipidaemia is not only influenced by HIV but is also strongly driven the exposure to PI-based regimens, highlighting the importance of switching to non-PI based therapies such as ﻿dolutegravir, which do not affect lipid composition (Quercia et al., 2015; Bagella et al., 2019).

To assess a possible link between the observed dyslipidemia and blunted immune response to vaccines in CLWH, Spearman’s rank correlation test was used to analyse these parameters. We did not find any association between lipid levels and IgG antibody responses to measles and pertussis. To further elucidate the link between these two phenomena, future studies should further interrogate the relatedness between systemic metabolic disorders and immune dysregulation (such as T-cell senescence), since recent data indicate that lipid-containing tissues of HIV+ subjects are enriched with highly senescent CD8^+^ T-cells (Godfrey et al., 2019), whose frequency is associated with CVD risk (Yu et al., 2016).

Our study was limited by its cross-sectional design; hence no causal inferences can be made. Moreover, lack of clinical CVD measurements limited correlating the observed CVD risk factors and immunoscenescence with clinical outcomes. The overall effect of ART on dyslipidemia and reduced vaccine-induced antibody responses could also not be elucidated in this study, since we unable enroll HIV+ART naïve comparator group due to an excellent rollout of ART in all diagnosed HIV+ Tanzanian children. Nonetheless, the main strength of our study was in matching the study groups to address confounding factors. The HIV+ and HIV- children were not only matched by age and sex, but also had similar BMI and blood pressure measurements (which may also influence the CVD outcomes). This allowed us to identify the extent of dyslipidemia and alteration of vaccine-induced humoral response in virologically controlling young CLWH in our setting.

The findings of this well-matched study complement those of earlier studies and strengthens the findings that dyslipidaemia driven by low HDL-c and hypertriglyceridemia is prevalent even in treated HIV+ African children with controlled viremia, suggesting that predisposition for CVD can begin much earlier in childhood, especially for persons living with HIV. High concentrations of triglycerides in CLWH were also associated with protease-based therapy, which could further increase CVD risk. Moreover, anti-measles and anti-pertussis toxin IgG titers were substantially reduced in vaccinated HIV+ children, suggesting a compromised humoral response to measles and pertussis. Future studies should monitor the identified CVD risk factors and immune-senescence prospectively, in association with CVD clinical outcomes. Monitoring of these events will be a prerequisite for improving care of individuals who acquired HIV at an early age, which will: i) prevent the premature onset of non-communicable diseases, in particular cardiovascular and metabolic ones and; ii) prevent the accelerated senescence of their immune system and therefore the susceptibility to preventable diseases.

## Data Availability Statement

The raw data supporting the conclusions of this article will be made available by the authors, without undue reservation.

## Ethics Statement

The studies involving human participants were reviewed and approved by Mbeya Medical Research and Ethics review Committee. Written informed consent to participate in this study was provided by the participants’ legal guardian/next of kin.

## Author Contributions

﻿All authors contributed to manuscript writing. WM, IM, FN, CN, LM, PM, and MC conceived, designed and conducted the study. PM and MC supervised the project. WM, WO, PA, and MC participated in data curation and statistical analysis. All authors contributed to the article and approved the submitted version.

## Funding

This work was supported by the University of Dar es Salaam (MCHAS-20022) and by Network Funds (PLAY HIGH and UAW project grants) from Ludwig Maximilians University of Munich (LMU)-Center for International Health (CIH), through DAAD/Exceed Program which is funded by the Ministry of Economic Efforts and Development of Germany (BMZ).

## Conflict of Interest

The authors declare that the research was conducted in the absence of any commercial or financial relationships that could be construed as a potential conflict of interest.

## Publisher’s Note

All claims expressed in this article are solely those of the authors and do not necessarily represent those of their affiliated organizations, or those of the publisher, the editors and the reviewers. Any product that may be evaluated in this article, or claim that may be made by its manufacturer, is not guaranteed or endorsed by the publisher.

## Supplementary Material

Supplementary Figure 1 No correlation between ART duration and IL-6 (pg/ml): Each dot represents an ART duration in months and IL-6 concentration pair for each individual volunteer. Statistical analysis was performed using the Spearman rank correlation test.

Supplementary Figure 2 Negative correlation between anti-measles IgG titers and age in HIV+ children: Each dot represents a pair of anti-measles IgG titer and age in years for each individual volunteer. Statistical analysis was performed using the Spearman’s rank correlation test.
