# Supplementary figures and images for: Altered Lipid Profiles and Vaccine Induced-Humoral Responses in Children Living With HIV on Antiretroviral Therapy in Tanzania
Source: Front Cell Infect Microbiol. 2021 Nov 9;11:721747. doi: 10.3389/fcimb.2021.721747 (PMC8630663; doi:10.3389/fcimb.2021.721747)

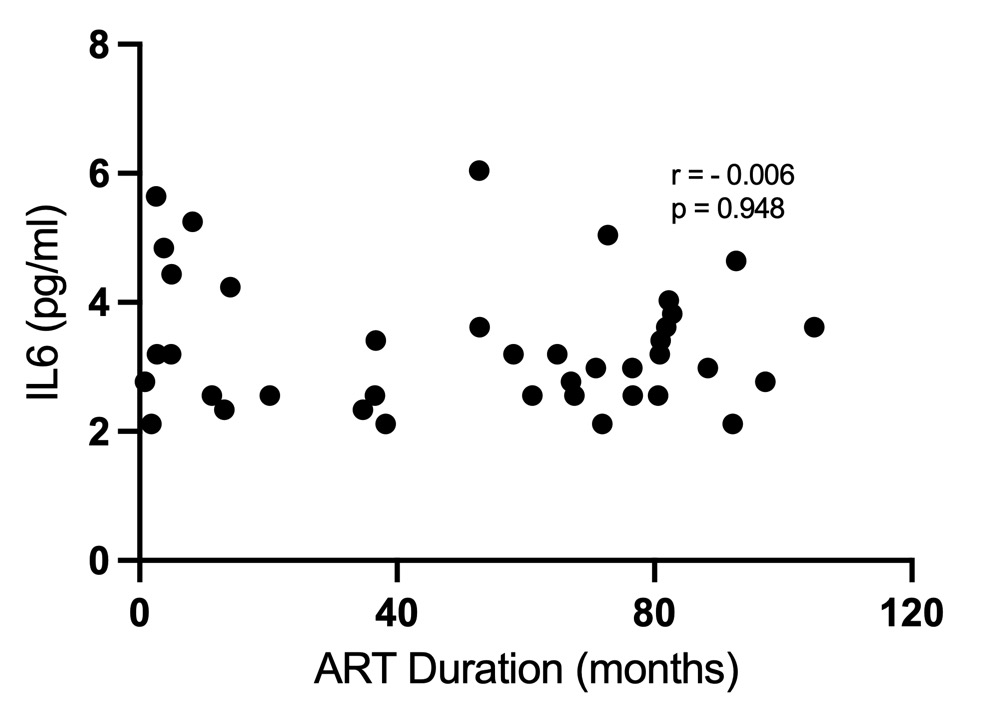

Supplement: Supplementary Figure 1 — No correlation between ART duration and IL-6 (pg/ml): Each dot represents an ART duration in months and IL-6 concentration pair for each individual volunteer. Statistical analysis was performed using the Spearman rank correlation test. [file Image_1.jpg]

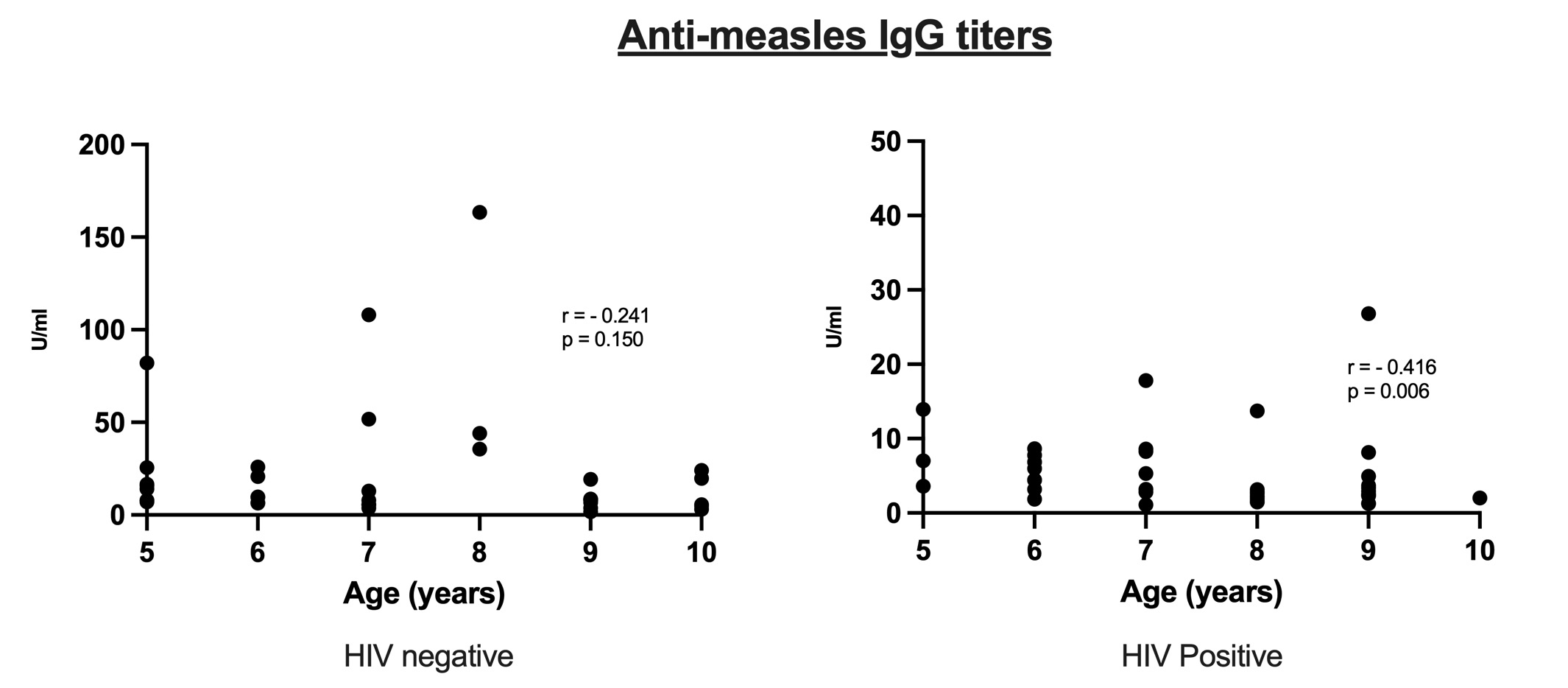

Supplement: Supplementary Figure 2 — Negative correlation between anti-measles IgG titers and age in HIV+ children: Each dot represents a pair of anti-measles IgG titer and age in years for each individual volunteer. Statistical analysis was performed using the Spearman’s rank correlation test. [file Image_2.jpg]
