# Supplementary material for: Altered Lipid Profiles and Vaccine Induced-Humoral Responses in Children Living With HIV on Antiretroviral Therapy in Tanzania
Source: Front Cell Infect Microbiol. 2021 Nov 9;11:721747. doi: 10.3389/fcimb.2021.721747 (PMC8630663; doi:10.3389/fcimb.2021.721747)
Supplement: Supplementary file 3 [file Table_1.docx]

**Supplementary table 1: Clinical chemistry reference values for serum samples established at NIMR-MMRC**

|  | **Male** | **Female** |
| --- | --- | --- |
| **Analyte** | Range(mmol/L) |  |
| Triglycerides | 0.38-2.18 | 0.39-2.88 |
| HDL-c | 1.10-2.10 | 1.10-2.10 |
| LDL-c | 1.10-4.30 | 1.10-4.30 |
| Cholesterol | 2.5-5.5 | 2.8-5.5 |

For each analyte, values out of the specified range are considered abnormal
